# Possibilities of Using the New *Lactiplantibacillus plantarum* EK11 Strain as a Starter Culture for the Fermentation of the Fruiting Bodies of Edible Mushrooms

**DOI:** 10.3390/foods14162833

**Published:** 2025-08-15

**Authors:** Ewa Jabłońska-Ryś, Krzysztof Przygoński

**Affiliations:** 1 Department of Plant Food Technology and Gastronomy, University of Life Sciences in Lublin, Skromna 8, 20-704 Lublin, Poland; 2 Food Concentrates and Starch Products Department, Prof. Waclaw Dabrowski Institute of Agricultural and Food Biotechnology—State Research Institute, Starołęcka 40, 61-361 Poznań, Poland; krzysztof.przygonski@ibprs.pl

**Keywords:** *Agaricus bisporus*, *Pleurotus ostreatus*, *Lentinula edodes*, *Lactiplantibacillus plantarum* EK11, lactic fermentation, fermented mushroom, basic components, biogenic amines, thiamine, riboflavin

## Abstract

Three popular cultivated mushroom species (*Agaricus bisporus*, *Lentinula edodes*, and *Pleurotus ostreatus*) were biopreserved through a directed lactic acid fermentation process. *Lactiplantibacillus plantarum* strain EK11 obtained from *A. bisporus* fruiting bodies subjected to spontaneous lactic acid fermentation was used as a starter culture. Regardless of the mushroom species, the pH value on experimental day 7 was ≤3.75, which guarantees the shelf life of fermented products; however, it decreased to 3.51–3.6 during refrigerated storage. The number of lactic acid bacteria in the final products exceeded 7 log colony forming units per mL. The fermentation process significantly reduced the caloric value and the digestible carbohydrate content, regardless of the mushroom species. It also reduced the protein content in the *P. ostreatus* and *L. edodes*. The protein in all the analyzed samples was composed of all essential amino acids, with the dominance of glutamic and aspartic acids responsible for the umami flavor. The fermentation process significantly improved the fatty acid profile, increasing the proportion of polyunsaturated fatty acids in the *P. ostreatus* and *L. edodes*. The fermented mushrooms contained significantly lower amounts of thiamine and riboflavin than the fresh ones, except for *L. edodes*, where the vitamin B1 content was unchanged. The starter used did not have the ability to synthesize biogenic amines. The fermented mushrooms achieved organoleptic scores ranging from 6.83 to 8.04 on a 9-point scale. *L. plantarum* strain EK11 can be regarded as a suitable starter culture for lactic acid fermentation of mushrooms.

## 1. Introduction

*Agaricus bisporus* (button mushroom), *Lentinula edodes* (shiitake), and *Pleurotus* spp., especially *Pleurotus ostreatus* (oyster mushroom), are the most popular species of cultivated mushrooms in Poland and worldwide [1,2]. These mushrooms are readily available as fresh products throughout the year, but are also processed and preserved. Freezing, canning, and drying are used for long-term preservation [1,2,3,4,5,6]. However, some of these methods, particularly pasteurization (marinades) or sterilization (salt brine-preservation), can considerably reduce the nutritional value of mushrooms [3,4]. An alternative may be the lactic acid fermentation process as a biological preservation method. Sivamaruthi et al. [7] emphasize that fermentation of mushrooms is an effective and inexpensive preservation method. It is mainly popular in Eastern Europe and Southeast Asia [1,8,9], although not on an industrial scale, as reported by Bartkiene et al. [10]. In Poland, mushrooms are currently fermented as part of household manufacture, but this method was widely used in the mid-20th century food industry for lactic acid fermentation of mainly *Lactarius deliciosus* and *Boletus edulis* mushrooms. During harvest seasons, this method ensured quick and inexpensive utilization of surplus raw materials [9]. Similarly, industrial processing of mushrooms was popular in the former Soviet Union and Czechoslovakia, but has now been replaced by other preservation methods [9,11]. As indicated by Sangeeta et al. [12], scaling up the mushroom fermentation process can offer numerous opportunities for innovation, product diversification, and sustainable development in various branches of industry.

For several years, the fermentation of edible mushroom fruiting bodies has been sporadically reported in the scientific literature. In all studies, mushrooms undergo directed lactic acid fermentation [7,9]. In the case of this raw material, the use of lactic acid bacteria (LAB) starter cultures is necessary, primarily due to the blanching process, which is one of the mushroom pretreatment steps. This short hydrothermal treatment is essential for removal of air from the raw material and achievement of anaerobic conditions optimal for LAB growth, inactivation of enzymes, and improvement of the purity and microbiological quality of mushroom fruiting bodies [7,9]. Authors of studies on the lactic acid fermentation of mushrooms used exclusively allochthonous strains as starter cultures. *Lactobacillus plantarum* (currently *Lactiplantibacillus plantarum*), dedicated primarily to the fermentation of plant-based materials, was used most commonly. *L. delbrueckii* subsp. *bulgaricus*, *L. delbrueckii* subsp. *delbrueckii*, *L. brevis*, *L. casei*, *L. helveticus*, *L. pentosus*, *Streptococcus lactis*, *Lactococcus lactis*, *Leuconostoc mesenteroides* and *Propionibacterium freudenrechii* were used effectively as well [7,9]. Mushrooms were also successfully fermented by *L. plantarum* strain 299v, whose probiotic properties are widely documented [13,14].

Microbial strains used as starter cultures are selected according to their biochemical, physiological, and genetic characteristics. However, this method for food production leads to the loss of traditional flavors and uniformity in the taste of products, regardless of the country or region [15]. The so-called endogenous (autochthonous) strains of lactic acid bacteria present in raw materials determine the characteristic sensory properties of fermented products. The use of these microorganisms as starters for the production of fermented edible mushrooms may also bring other benefits. Lactic acid bacteria are better adapted to the raw material, which ensures better utilization of the characteristic mushroom sugars as a nutrient and, consequently, faster and more controllable fermentation [15,16,17]. The objective of this study was to assess the potential of the *L. plantarum* EK11 strain, which was isolated from *A. bisporus* fruiting bodies subjected to spontaneous lactic acid fermentation and described in previous studies [16,18], to be used as a starter culture in the process of lactic acid fermentation of the fruiting bodies of three most popular cultivated mushroom species. Additionally the quality of the final products was assessed.

## 2. Materials and Methods

### 2.1. Raw Materials

Fruiting bodies of the white variety of *Agaricus bisporus*, *Pleurotus ostreatus*, and *Lentinula edodes* were analyzed in the study. The mushrooms were purchased from a local market and processed immediately after the purchase (maximum after 12 h from harvest). The new lactic acid bacteria *Lactiplantibacillus plantarum* EK11 strain, i.e., one of the isolates obtained from fermented fruiting bodies of *A. bisporus*, described in previous research [16,18], and deposited to the GenBank with the accession number MW040528.1, was the starter culture. LAB were propagated twice in MRS broth (Biocorp, Warsaw, Poland) and incubated (TK-2, Cabrolab, Warsaw, Poland) overnight at 30 °C. Microbial cells were centrifuged (MPW 350-R, MPW, Warsaw, Poland) at 1400 *g* for 10 min, harvested, and washed twice in sterile 0.9% NaCl (P.O.Ch., Gliwice, Poland) before inoculation.

### 2.2. Preparation of Fermented Mushrooms

**Figure 1:**
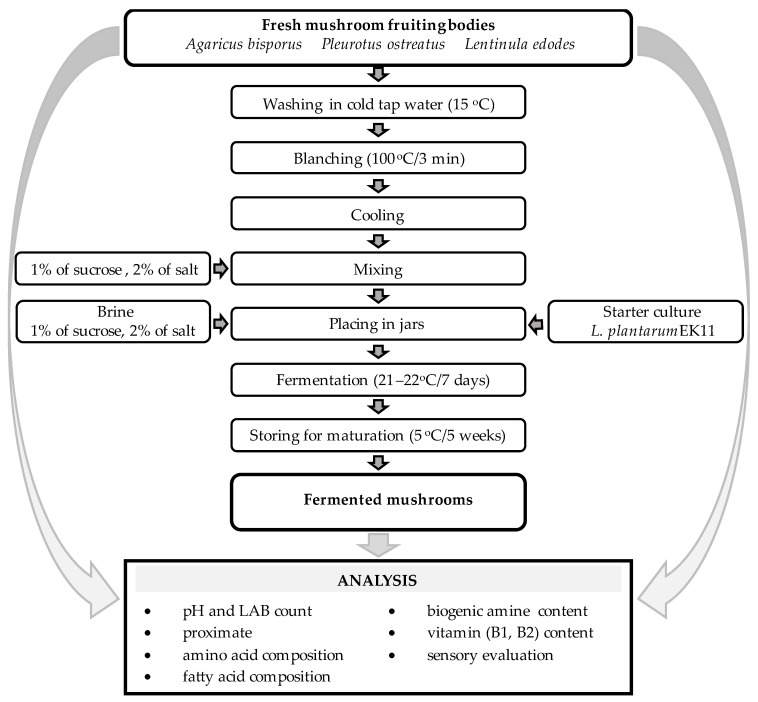
Experimental scheme.

The fermentation procedure followed that used in a previous study [19] with some modifications. *A. bisporus* and *L. edodes* fruiting bodies with a diameter of 3.5–4.5 cm and short-trimmed stipes were used in the fermentation process. The mushrooms were cleaned thoroughly to remove substratum debris and rinsed in cold tap water with a temperature of approximately 15 °C. The *A. bisporus* and *L. edodes* mushrooms were left whole, while the *P. ostreatus* mushrooms with a diameter above 4 cm were cut in half with a knife. Next, the mushrooms were subjected to blanching in boiling water for 3 min. When cooled, 2% (*w*/*w*) NaCl and 1% (*w*/*w*) sucrose were added to the samples. The mushrooms were divided into 380 g portions and transferred into 500 mL glass jars. Then, 120 mL of a solution containing 2% NaCl and 1% sucrose was added. Afterwards, the starter culture (*L. plantarum* EK11) was inoculated with 10^7^ CFU (colony-forming unit) per gram of fermented material and the jars were closed. The initial value of pH was approximately 6.7. The lactic fermentation was maintained for 7 days at 21–22 °C, after which the fermented mushrooms were stored in the same sealed intact jars at 5 °C for 5 weeks for maturation. The fermentation process was performed in triplicate. The samples of fresh and fermented mushrooms were stored at −80 °C until the basic components: fatty acids, amino acids, biogenic amines, and vitamins were determined. The other analyses were performed on an ongoing basis. A diagram illustrating the research process is shown in Figure 1.

### 2.3. Microbiological Analysis

The LAB count in the brine was measured during the fermentation in compliance with PN-ISO 15214:2002 [20]. The analyses were carried out on days 0, 7, 14, and 42 of the experiment. The bacterial counts were expressed as the log CFU/mL of the sample.

### 2.4. Determination of pH

A digital pH meter (Seven Compact S210, Mettler Toledo, Greifensee, Switzerland) was used to measure the pH value in the brine during the fermentation process. These analyses were conducted on days 0, 7, 14, and 42 of the experiment.

### 2.5. Basic Composition of Fresh and Fermented Mushrooms

The basic components: moisture, protein, fat, ash, and total dietary fiber of fresh and fermented mushrooms, were determined using the methods thoroughly described by Grdeń and Sołowiej [21]. The amount of digestible carbohydrates was obtained by subtraction of the sum of these basic ingredients from 100. The amount of digestible carbohydrates was calculated according to the following equation:

(1) digestible carbohydrates (%) = 100 − (% moisture + % protein + % fat + % ash + % dietary fiber).

Factor 4.38 [22] was used to estimate the level of raw protein in the mushroom samples to account for the substantial chitin content. Based on the amount of the basic components, the energy value was calculated according to the following equation [23]:

(2) energy (kcal) = 4 (g protein + g carbohydrate) + 9 (g fat) + 2 (g dietary fiber).

All measurements were performed in triplicate and expressed as a mean ± standard deviation (SD). The nutritional value was expressed in % of dry weight (DW) and the energy value was expressed in kcal per 100 g DW.

### 2.6. Amino Acid Composition

Samples for determination of the amino acid (AA) were prepared according to the methodology described by Grdeń and Sołowiej [21]. The analysis of amino acids was performed using an AAA400 amino acid analyzer (Ingos, Prague, Czech Republic) equipped with an Ostion LG ANB ion exchange column (0.37 cm × 45 cm) kept at 60 °C and 74 °C. The apparatus detects amino acids with the use of ninhydrin (detection reagent). Detection was carried out with spectrophotometry at 570 nm for all amino acids (except for 440 nm used for detection of proline). Four buffers with pH values of 2.6, 3.0, 4.25, and 7.9 were used for separation. After separation of amino acids, the column was regenerated with 0.2 N NaOH. The analysis was performed in triplicate and expressed as a mean ± standard deviation (SD). The concentrations of amino acids were expressed in mg/g DW.

### 2.7. Fatty Acid Composition

Samples for determination of fatty acids were prepared according to the methodology described by Grdeń and Sołowiej [21]. Chromatographic separation was performed using a Varian 450-GC gas chromatograph with Galaxie Chromatography Data System software (Varian Inc., Walnut Creek, CA, USA). The following parameters of the separation process were used: stationary phase: Select Biodiesel for FAME Fused Silica; column oven: initial temp. 100 °C, final temp. 240 °C; FID detector temp. 270 °C. carrier gas: helium; carrier gas flow rate: 1.5 mL/min. The analysis was performed in triplicate and expressed as a mean ± standard deviation (SD). The fatty acid profile was presented as a percentage.

### 2.8. Determination of Biogenic Amines

Biogenic amines (BAs) were extracted from the mushroom samples as in Rabie et al. [24] with some modifications. Three-gram samples (fresh and fermented mushrooms) were homogenized with 10 mL of 10% (*w*/*v*) trichloroacetic acid (TCA) in a homogenizer (IKA T10 basic ULTRA-TURRAX, Staufen, Germany) and shaken for 1 h in an orbital shaker (DragonLab, SK-330-Pro, Beijing, China). Next, the samples were centrifuged at 1957 *g* for 15 min at 4 °C (MPW-350R, Warsaw, Poland). The supernatant was filtered through a 0.22 μm membrane filter (Alfachem, Poznań, Poland) and dosed onto the amino acid analyzer. The BA analysis was performed with the use of an AAA400 amino acid analyzer (Ingos, Prague, Czech Republic) equipped with an Ostion LG ANB ion exchange column (7 cm × 0.37 cm) kept at 76 °C. Separation was performed by stepwise gradient elution using Na^+^/K^+^ citric buffers. Detection was carried out with the spectrophotometry method at 570 nm after postcolumn derivatization with ninhydrin. The following operating parameters were employed: eluent and reagent flow rates of 0.50 and 0.33 mL/min, respectively; reactor temperature of 96 °C; total run time of 97 min. The results of the determination of biogenic amines (histamine, cadaverine, putrescine, spermine, spermidine, tyramine, and agmatine) were confirmed through comparison of the retention times and peak areas of the particular biogenic amine standards (Sigma Aldrich, Steinheim, Germany) with those of the components present in the samples. The analysis was carried out in triplicate and the results were expressed as a mean ± standard deviation (SD). The concentrations of biogenic amines were reported as mg/g DW.

### 2.9. Determination of Thiamine and Riboflavin

Thiamine was extracted from the food matrix after enzymatic (takadiastase) and acidic (0.1 M H_2_SO_4_) hydrolysis. The extract obtained was subjected to a reaction converting thiamine into the thiochrome form, which was extracted from the reaction mixture with isobutyl alcohol. Alcohol with thiochrome was dissolved in the mobile phase used for HPLC in a ratio of 1:9. The samples were subjected to high-performance liquid chromatography (HPLC) with the use of a DIONEX chromatograph combined with a P680 pump (DIONEX, Sunnyvale, CA, USA), an RF-2000 fluorescence detector (DIONEX, Sunnyvale, CA, USA), an ASI-100 autosampler (DIONEX, Sunnyvale, CA, USA), and a TC-100 thermostat (DIONEX, Sunnyvale, CA, USA). The chromatographic separation was carried out using a ThermoScience C18 column (250 mm × 4.6 mm, 5 µm). Then, 20 µL of each sample was dispensed onto the column. The mobile phase consisted of methanol and ultrapure water (7:3, *v*/*v*) with isocratic elution at a flow rate of 1 mL/min. The detection was performed at EX excitation and EM emission wavelengths of 360 nm and 440 nm, respectively.

Riboflavin was extracted from the food matrix after enzymatic (takadiastase) and acidic (0.1 M H_2_SO_4_) hydrolysis. The obtained extract was filtered (0.45 µm) and the filtrate was injected onto a chromatographic column. The riboflavin content was determined according to EN 14152:2014 [25]. The high-performance liquid chromatography (HPLC) analysis was performed using a DIONEX chromatograph combined with a P680 pump (DIONEX, Sunnyvale, CA, USA), an RF-2000 fluorescence detector (DIONEX, Sunnyvale, CA, USA), an ASI-100 autosampler (DIONEX, Sunnyvale, CA, USA), and a TC-100 thermostat (DIONEX, Sunnyvale, CA, USA). A Supelco Discovery HS C18 column (100 mm × 4.6 mm, 5 µm) was used for chromatographic separation. Then, 20 µL of each sample was dispensed onto the column. The mobile phase comprised methanol and ultrapure water (6:4, *v*/*v*) with isocratic elution at a flow rate of 1 mL/min. The detection was performed at EX excitation: 450 nm and EM emission: 520 nm. The analysis was performed in triplicate and expressed as a mean ± standard deviation (SD). The vitamin concentrations were reported as mg/100 g DW.

### 2.10. Sensory Evaluation

A 9-point hedonic scale (where 1 = extremely dislike, 5 = neither like nor dislike, 9 = extremely like) was used for the evaluation of the sensory parameters of the fermented mushrooms. A panel of 23 untrained consumers aged 20 to 54 (13 females and 10 males) assessed the color, aroma, taste, and texture of the fermented mushrooms. The samples were coded with three random digits. The serving order was randomized as well. The research was approved by the ethics committee. The study results were expressed as mean value ± standard deviation (SD).

### 2.11. Statistical Analysis

The results were subjected to statistical analyses performed using the STATISTICA 13.1 program (StatSoft, Cracow, Poland) and Tukey’s HSD test for the analysis of variance (ANOVA) to estimate the significance of the differences between the mean values at *p* < 0.05.

## 3. Results and Discussion

### 3.1. Changes in pH During Mushroom Fermentation

**Figure 2:**
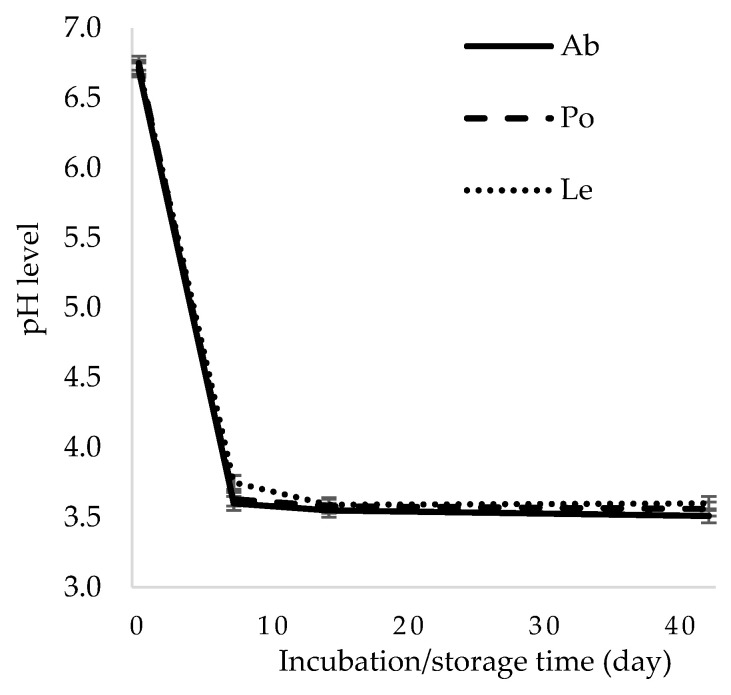
Changes in the pH value in the fermented mushrooms. Ab—*Agaricus bisporus*, Po—*Pleurotus ostreatus*, Le—*Lentinula edodes*.

A decrease in the pH parameter is an important indicator corroborating the proper course of lactic acid fermentation, as it indirectly indicates LAB colonization of the environment and production of lactic acid, i.e., the primary determinant of the stability of fermented products. The pH value on day 7 of the experiment ranged from 3.6 to 3.75 in all the samples of the *A. bisporus* and *L. edodes*, respectively (Figure 2). During the refrigerated storage, the pH of the fermented mushrooms decreased slightly, reaching a level between 3.51 and 3.6 in the *A. bisporus* and *L. edodes*, respectively. These results are similar to the findings reported in previous studies on *A. bisporus* and *P. ostreatus* fermented with the use of *L. plantarum* 299v, *L. plantarum* EK3, *L. plantarum* Ib, *L. casei* Lby, and *L. helveticus* K1Lb [13,14,17,19]. As shown by Steinkraus [26], a pH value below 4 ensures stability of fermented vegetables and maintains anaerobic conditions.

### 3.2. Changes in Microbial Abundance During Fermentation

**Figure 3:**
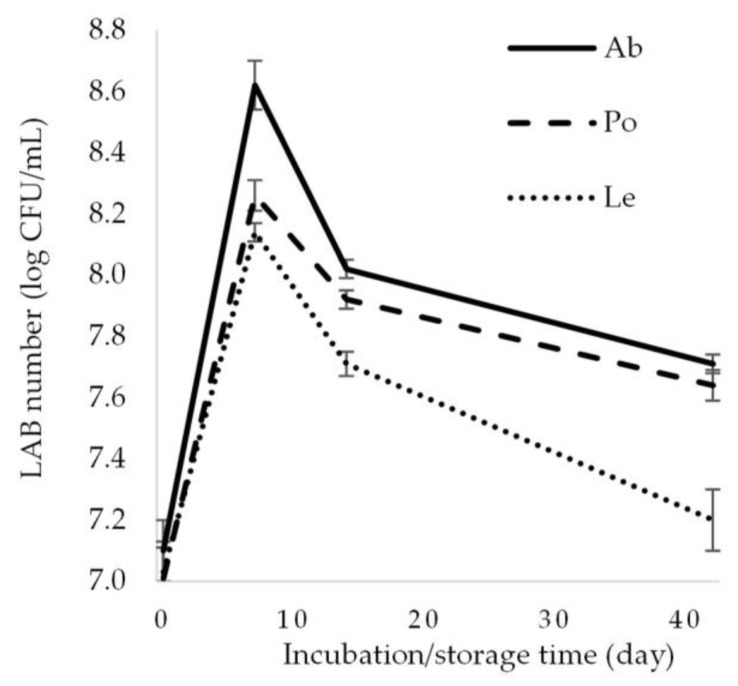
Changes in the LAB count in the fermented mushrooms. Ab—*Agaricus bisporus*, Po—*Pleurotus ostreatus*, Le—*Lentinula edodes*.

The LAB count introduced with the starter was 7 log CFU/mL. On day 7 of the experiment, this value increased to 8.62, 8.26, and 8.14 log CFU/mL in the samples of fermented *A. bisporus*, *P. ostreatus*, and *L. edodes* mushrooms, respectively (Figure 3). These values declined during the refrigerated storage of the fermented samples; nevertheless, the LAB count exceeded 7 log CFU/mL in each product, regardless of the mushroom species, in the final phase of the experiment (day 42). The highest LAB count was determined in the fermented *A. bisporus* (7.71 log CFU/mL). Noteworthy, the *L. plantarum* EK11 strain used in the experiment had been isolated from *A. bisporus* fruiting bodies subjected to spontaneous lactic acid fermentation [16], which may explain the best adaptation of the strain to this nutritional matrix. This was also evidenced by the highest lactic acid production, as indicated by the lowest pH value in the fermented *A. bisporus*. Reduced LAB abundance is caused by lower availability of carbohydrates (nutrient source) and the inhibitory effect of low pH associated with increased lactic acid production. Similar LAB counts in the final product were demonstrated, e.g., in *A. bisporus* fermented by *L. plantarum* 299v, *L. plantarum* EK3 [14], *Lacticaseibacillus casei* 210, and *L. plantarum* 135 [10] and in *P. ostreatus* fermented by *L. plantarum* [27] and *L. pentosus* [3].

### 3.3. Proximate Composition

#### 3.3.1. Protein

The nutritional value of mushrooms is mainly determined by the amount of protein, which is on average 20–40% DW. Mushroom protein is characterized by high digestibility levels reaching 90% in some species [28].

The fresh (raw) mushroom fruiting bodies varied significantly in their protein content. The highest protein content was found in the fresh *L. edodes* fruiting bodies, while the lowest amount was determined in the *P. ostreatus* (Table 1). The fermentation process influenced the protein content differently, depending on the mushroom species. In the case of the *A. bisporus*, the initial protein content (35.8% DW) was unchanged. The protein content in the *P. ostreatus* and *L. edodes* mushrooms decreased significantly from 29.5% and 37.5% DW to 25.5% and 26% DW, respectively. The decline in the protein content may be a result of soluble protein leaching into the water during the blanching process or into the brine during the fermentation process. Amino acids and other nitrogen compounds can be utilized by LAB as well. Proteolysis is an important metabolic activity of lactic acid bacteria, as they are incapable of the synthesis of many amino acids or nucleic acids and must hydrolyze proteins and peptides present in fermented raw materials [29]. This process (proteolysis) can largely contribute to increased protein digestibility from fermented products, compared to fresh raw materials. This is crucial in the case of mushrooms, as they are considered a valuable source of protein, especially in vegan diets.

In some cases, the protein content in mushrooms can increase during fermentation. As reported by Ogidi and Agbaje [30], fermented *P. ostreatus* contained significantly higher amounts of protein than fresh materials. This increase was attributed to the release of proteins bound to cell wall polysaccharides through the action of LAB proteolytic enzymes.

#### 3.3.2. Fat

The total fat content in the fruiting bodies of edible mushrooms is relatively low. In the present study, the *A. bisporus* fruiting bodies had the highest fat content (3.4% DW) (Table 1). This is consistent with available literature data [1,22,31]. The lactic acid fermentation process had a varying effect on the fat content, depending on the mushroom species. A significant increase in the fat content from 3.4 and 2.3% DW to 4.2 and 3.1% DW was observed in the *A. bisporus* and *L. edodes*, respectively. In turn, the fermented *P. ostreatus* fruiting bodies contained significantly lower amounts of fat than the fresh mushrooms. Similarly, Odigi and Agbaje [30] reported a decline in the fat content from 0.8 to 0.5% DW in fermented *P. ostreatus* samples. The percentage increase in the fat content in the dry matter of the samples may be related to the decrease in the content of other components, i.e., carbohydrates, proteins, or other water-soluble substances.

#### 3.3.3. Digestible Carbohydrates

Carbohydrates constitute the largest portion (35–70%) of edible mushroom dry matter [1,32], but most carbohydrates are indigestible and are part of dietary fiber. The amounts of digestible carbohydrates in the analyzed mushroom species ranged from 8.5 to 17.2% DW in the *P. ostreatus* and in the *A. bisporus*, respectively (Table 1). Despite the addition of sucrose in accordance with the recipe, the content of digestible carbohydrates in the fermented mushroom samples was several times lower than in the fresh mushrooms and ranged from 1.2 to 2.3% DW in the *L. edodes* and *A. bisporus*, respectively. Carbohydrates are the main carbon source for LAB, but these compounds can be lost during the blanching process or through the contact with the brine during fermentation. Soluble carbohydrates then leach into water. Previous studies [14] demonstrated that 100% of fructose, glucose, and sucrose, which were present in small amounts in fresh mushroom fruiting bodies (8.74, 9.72, and 53.5 mg/100 of fresh weight (FW), respectively), were lost during blanching. The trehalose and mannitol content also decreased significantly. Only the ribose content did not exhibit any significant changes. Li et al. [33] studied the effect of blanching on the chemical composition of *L. edodes* and confirmed that hydrothermal processing contributed to significant losses of soluble sugars and polyols. This indicates that blanched mushrooms do not contain a sufficient amount of this readily available carbon source. As reported by Zheng et al. [27], *P. ostreatus* fruiting bodies contain lower amounts of fermentable saccharides (0.68%) than their levels in cabbage (1.5–2.2%) or cucumber (2%). Hence, most researchers investigating the mushroom fermentation process suggest the necessity to add 1–3% of sugar, such as sucrose or, alternatively, glucose or fructose [9].

#### 3.3.4. Total Fiber

Mushrooms are a fiber-rich raw material. They consist of water-insoluble fibers, such as chitin, and soluble fibers, primarily β-glucans and chitosan, with the predominance of insoluble fibers [22,34]. The fiber content in the fresh fruiting bodies of the analyzed mushrooms ranged from 33.3% DW (*A. bisporus*) to 49.9% DW (*P. ostreatus*) (Table 1). The fermentation process induced changes in the fiber content, but they were statistically insignificant in the case of the *A. bisporus* and the *P. ostreatus*. A significant increase in the fiber content was recorded in the fermented *L. edodes* samples. Fiber losses are probably related to the soluble fraction, which can leach into the water during the blanching process or into the brine during fermentation or refrigerated storage. As reported by Odigi and Agbaje [30], certain fiber fractions can be utilized by LAB to produce other metabolites. An increase in the percentage of fiber in fermented mushrooms can probably be explained by a decrease in the content of other dry matter components, e.g., carbohydrates. Mushroom fibers, especially β-glucans, are highly valuable dietary components. These compounds have been shown to have numerous health-enhancing properties, e.g., anticancer, antioxidant, anti-inflammatory, antibacterial, antiviral, antiallergic, immunomodulatory, and many other effects [35,36]. As demonstrated by Cerletti et al. [35], *A. bisporus*, *P. ostreatus*, and *L. edodes* fruiting bodies contain 8.6, 24.2, and 20 g of β-glucans per 100 g DW, respectively.

#### 3.3.5. Ash

The ash content in the fresh mushroom fruiting bodies varied significantly (Table 1), with the highest amount in the *A. bisporus* (10.4% DW) and the lowest content (7.4% DW) in the *L. edodes* samples. This is consistent with the range (5–12% DW) reported for various edible mushroom species by Kalač [22]. An over two-fold increase in the ash content was found in the fermented mushroom samples. A significant increase in the content of this component in the process of lactic acid fermentation of *P. ostreatus* fruiting bodies was also reported by Ogidi and Agbaje [30]. Similar findings were shown by Sultana et al. [37] and Chakriya et al. [38], who demonstrated a several-fold increase in the ash content in vegetables exposed to the lactic acid fermentation process. The ash content is closely associated with the mineral content. As suggested by Bello and Akinyele [39], the addition of salt has the greatest impact on the increase in the mineral content in fermented mushrooms.

#### 3.3.6. Energy Value

Due to their low fat content, relatively low digestible carbohydrate content, and high fiber content, mushrooms are generally considered low-calorie foods. As shown in Table 1, the fresh *A. bisporus* had the highest caloric value (309 kcal/100 g DW), whereas the lowest value was determined for the *P. ostreatus* (278.8 kcal/100 g DW). The fermentation process had a significant impact on the caloric value of the mushrooms, i.e., it was significantly reduced in the fermented material. This is mainly related to the metabolic activity of LAB, decomposition of sugars, and soluble component leaching into the water during the blanching process or into the brine.

### 3.4. Amino Acid Composition

Proteins are made up of 20 amino acids (AA). Nine amino acids, i.e., leucine, isoleucine, threonine, methionine, phenylalanine, lysine, valine, tryptophan, and histidine, are classified as essential amino acids (EAA), as they are not synthesized by human cells. Therefore, these amino acids must be acquired from nutrients [40]. Proteins from edible mushrooms usually have a complete profile of essential amino acids; hence, mushrooms can be a very good meat substitute [41,42]. The amino acid composition in mushrooms, including the presence of essential amino acids, is influenced by cultivation methods, growth conditions, and mushroom species [43]. The fresh fruiting bodies of the analyzed mushrooms differed significantly in their amino acid composition (Table 2). Glutamic and aspartic acids were the most abundant amino acids in each of the analyzed species. The highest content of aspartic acid was found in the fresh *A. bisporus* fruiting bodies (37.57 mg/g DW), and the highest content of glutamic acid was determined in the *L. edodes* fruiting bodies (56.28 mg/g DW). As reported by Bach et al. [44], these compounds are the dominant amino acids in *A. bisporus* (Champignon and Portobello), *A. brasiliensis*, *L. edodes*, *P. ostreatus* (white and black oyster), *P. djamor*, *P. eryngii*, and *Flamulina velutipes*. This may be associated with the role of these two AAs as precursors of other AAs [45]. Glutamic and aspartic acids are responsible for the characteristic umami flavor of mushrooms [46]. The analyzed mushroom samples contained a complete set of exogenous amino acids. The concentration range of each EAA in all the unprocessed mushroom samples (mg/g DW) was as follows: threonine (5.98–13.32), valine (5.62–12.51), methionine (7.29–7.89), isoleucine (6.04–16.79), leucine (8.39–18.37), phenylalanine (9.1–14.01), histidine (3.31–7.25), lysine (6.79–15.46), and tryptophan (1.44–6.13). The highest EAA content was exhibited by the *A. bisporus* fruiting bodies (111.15 mg total EAA/g DW), and the lowest amount was determined in the *P. ostreatus* fruiting bodies (54.57 mg total EAA/g DW).

The fermentation process altered the amino acid profile, and the type of changes depended on both the mushroom species and the AA type. In the case of *P. ostreatus*, the content of the majority of amino acids increased in the fermented samples, compared to the fresh mushrooms, with the exception of cysteic acid, methionine, and phenylalanine. The content of total AAs and EAAs increased by over 10% and over 12%, respectively, compared to the unprocessed *P. ostreatus* samples. The fermentation process resulted in a decrease in the content of all amino acids in the *A. bisporus* and *L. edodes* samples. The content of total AAs and EAAs in the fermented mushrooms decreased by over 22% and over 17%, respectively. The amount of total AAs and EAAs in the fermented *L. edodes* fruiting bodies declined by almost 36% and over 30%, respectively. The impact of lactic acid fermentation on the amino acid content was discussed in only one publication. Ogidi and Agbaje [30], who subjected *P. ostreatus* and *Termitomyces robustus* fruiting bodies to lactic acid fermentation, observed an increase in the content of almost all AAs, except glutamine, glycine, and histidine, in the fermented mushroom samples. The authors ascribed the increase in the amine content in the fermented mushrooms to the activity of LAB proteolytic enzymes. Chen et al. [47] fermented *L. edodes* mushrooms using various microbial species (*Saccharomyces cerevisiae*, *Aspergillus oryzae*, *Aspergillus niger*, and *L. plantarum*) and reported an increase in the free amino acid content in the fermentation liquid. They also found that the *L. plantarum*-induced fermentation contributed to the greatest increase in the content of substances responsible for the umami flavor. The AA content in processed mushrooms may be affected by processing methods, e.g., blanching. These compounds can leach from the mushrooms into the water during the hydrothermal treatment and into the brine during storage of the product. Jaworska et al. [48,49] analyzed the impact of various pre-treatment methods on the amino acid content in frozen and sterilized (canned) mushrooms. The losses of some amino acids amounted to 21% in the processed *A. bisporus* and as much as 39% in the *P. ostreatus*. During heat treatment, the side chains of some protein-bound amino acids react chemically with each other, which may result in changes in the composition of these amino acids. The decline in amino acid levels may also be associated with non-enzymatic browning reactions, which lead to the condensation of amine groups of protein amino acids with sugars and decomposition of amino acids during further transformations [48,49,50]. The fermentation process itself may also contribute to AA losses. As reported by Lee et al. [51], *L. plantarum* bacteria utilize amino acids present in the medium for their growth and lactic acid production.

### 3.5. Biogenic Amine Composition

Mushrooms are a protein-rich raw material (a source of amino acids—precursors of amines), but they are highly perishable. This may exert a significant impact on the content of biogenic amines (BAs) in mushroom raw material and products. In the cells of living organisms, these compounds perform important functions, but large contents of BAs, primarily histamine, supplied with food may have toxic effects [52,53]. Only spermidine out of the seven BAs analyzed was detected in the mushroom samples. Histamine and tyramine, which are generally considered the most toxic BAs, were not detected in any of the samples (Table 3).

In the fresh material, the highest amounts of spermidine were detected in the *A. bisporus*, and the lowest levels were determined in the *P. ostreatus* (2.56 and 0.69 mg/g DW, respectively). Spermidine is typically the most abundant polyamine of all BAs present in mushrooms [53,54]. This compound exerts excellent cardioprotective and neuroprotective effects, has anti-inflammatory activity, and protects stem cells against aging. Preclinical models have shown that dietary supplementation with spermidine significantly improves health and extends the lifespan [55,56]. The literature data on the content of this compound in mushrooms are inconsistent. For example, *A. bisporus* fruiting bodies may contain from 43.45 to as much as 4357.5 mg of spermidine per kg FW [54,57,58]. The content of BAs in mushrooms is determined by multiple factors, e.g., the maturity degree, harvest methods, conditions and duration of storage, and morphological part [54]. As shown by Reis et al. [59], in addition to spermidine present in the highest amounts (3.23–17.2 mg/100 g FW) in the fruiting bodies of *Agaricus bisporus, Lentinula edodes*, and *Pleurotus* spp., the mushrooms also contained agmatine (in every *Pleurotus* spp. and some *L. edodes* samples), phenylethylamine (in some *Pleurotus* spp. samples), and tyramine and tryptamine (in some mushroom species). Cadaverine and putrescine were present in trace amounts only in the *Pleurotus* spp. samples. Fermented products are at risk of high biogenic amine contents due to the presence of microorganisms and, specifically, their enzymes—decarboxylases [52]. Food subjected to spontaneous fermentation is at particular risk [60,61]. The fermented *A. bisporus* and *L. edodes* fruiting bodies exhibited significantly lower spermidine contents than the fresh mushrooms (Table 3). It is believed that BAs are thermally stable but may leach into water during the blanching process [62]. As demonstrated by Jabłońska et al. [17], blanched white and brown mushroom fruiting bodies were characterized by a significant decrease in the spermidine content, whereas no changes in the level of this compound were noted during lactic acid fermentation and refrigerated storage. Bartkiene et al. [10] showed similar spermidine contents in unfermented white and brown mushroom fruiting bodies and samples fermented using various LAB strains (180.0–222.3 mg/kg FW). Tyramine, with levels ranging from 75.1 to 87.6 mg/kg FW, was detected in some fermented mushroom samples. Pre-treatment applied before fermentation may result in an increase in the BA content in some cases. The application of ultrasonication before the fermentation process resulted in an average 15.3- and 2.4-fold increase in the total BA content in *Boletus edulis* and *Rozites caperata* samples, respectively, compared to samples subjected to the traditional heat treatment [63].

The inability to synthesize biogenic amines is a highly important characteristic of LAB used as starter cultures. As specified by the EFSA (European Food Safety Authority) guidelines, starter cultures should not be capable of synthesizing biogenic amines. The *L. plantarum* EK11 strain used in the present study did not cause an increase in the BA content in any of the samples.

### 3.6. Fatty Acid Composition

Mushrooms contain small amounts of fat, typically with a favorable ratio of unsaturated to saturated fatty acids [22]. The fatty acid (FA) content (% of the total fat content) in the mushroom samples is shown in Table 4. The highest share of total polyunsaturated fatty acids in the fresh mushrooms was exhibited by the *A. bisporus* fruiting bodies (64.32%), whereas the lowest amounts were detected in the *L. edodes* samples (26.71%). Similar proportions of total saturated fatty acids (SFAs), monounsaturated fatty acids (MUFAs), and polyunsaturated fatty acids (PUFAs) were found in the *P. ostreatus* fruiting bodies. Omega-6 fatty acids largely predominated in all the fresh mushroom samples. Linoleic acid (C18:2), determined as the sum of its *cis* and *trans* forms, was the dominant polyunsaturated fatty acid in the fresh mushroom material. It constituted from 26.33% (*L. edodes*) to as much as 63.07% (*A. bisporus*) of total FAs. Other PUFAs identified in the fresh mushroom samples included γ-linolenic acid (C18:3n6), present only in the *L. edodes* samples, α-linolenic acid (C18:3n3), present in the *P. ostreatus* and *L. edodes* mushrooms, cis-8,11,14-eicosatrienoic acid (C20:3n6), contained only in the *A. bisporus* samples, cis-5,8,11,14,17-eicosapentaenoic acid (C20:5n3), and cis-4,7,10,13,16,19-docosahexaenoic acid (C22:6n3), detected only in the *P. ostreatus* fruiting bodies. C18:1 acid, determined as the sum of oleic acid and elaidic acid, was the dominant MUFA, constituting from 7.61% (*A. bisporus*) to 30.79% (*P. ostreatus*). Other MUFAs identified in the fresh mushroom samples included palmitoleic acid (C16:1n7), cis-11-eicosenoic acid (C20:1n9), present only in the *L. edodes* fruiting bodies, and erucic acid (C22:1n9). Palmitic acid (C16:0) was the most abundant SFA, constituting from 16.65% to 23.65% of total FAs in the *A. bisporus* and *L. edodes*, respectively. The highest share of SFAs was detected in the *L. edodes* fruiting bodies. In addition to palmitic acid, this FA fraction primarily included stearic acid C18:0 (12.79%), capric acid C10:0 (7.40%), myristic acid C14:0 (6.89%), and lauric acid C12:0 (3.32%).

As reported by Kalač [22], linoleic and oleic acids account for over 2/3 of the FA share in edible mushrooms, and saturated palmitic acid is the third most common FA. A study conducted by Bartkiene et al. [10] has demonstrated that linoleic acid is the main FA present in *Agaricus bisporus* fruiting bodies (72.2–75.6%). Stojković et al. [64] reported a slightly lower FA amount, i.e., 43.87%. As shown by Das et al. [65], the maximum amount of linoleic acid was discovered in *Pleurotus ostreatus* (65.59%) > *Lentinula edodes* (58.23%) > *Agaricus bisporus* (40.33%). In turn, in *P. ostreatus* fruiting bodies, Ogidi and Agbaje [30] found the highest amounts of palmitoleic acid (21% of all FAs), while linoleic acid with a share of 10.6% was the fourth most abundant FA after linolenic (15.9%) and lauric (13%) acids. Linoleic acid has been reported to mitigate various health conditions through its anti-atherogenic, anti-inflammatory, anti-obesity, anticancer effects and its role in immunomodulation and osteosynthesis [66]. This compound is also a precursor of the attractive aroma of dried mushrooms [22].

The process of lactic acid fermentation caused significant changes in the fatty acid profile. The SFA content in the *P. ostreatus* and *L. edodes* mushrooms decreased from 33.61% and 60.56% to 22.20% and 37.11%, respectively. The MUFA content decreased in all the fermented mushroom samples, while the PUFA content increased significantly. In the *P. ostreatus* and *L. edodes* samples, the lactic acid fermentation process significantly improved the fatty acid profile, increasing the PUFA content from 33.37% and 26.71% to 55.63% and 54.62%, respectively. Ogidi and Agbaje [30] subjected *P. ostreatus* and *Termitomyces robustus* fruiting bodies to lactic acid fermentation and described different changes in the MUFA content. In the case of *P. ostreatus*, the fermentation process resulted in a significant increase in the content of this fatty acid fraction. As shown by Bartkiene et al. [10], changes in the fatty acid profile induced by fermentation are correlated significantly with the starter culture type and mushroom species. The comparison of two *A. bisporus* varieties (white and brown) revealed a significant increase in the PUFA content and a decrease in the SFA and MUFA content of the fermented brown mushrooms, regardless of the starter culture used. The authors attributed these changes in the fatty acid profile to the differences in the impact of lactic acid bacteria, whose metabolism may favor lipid oxidation during the fermentation process or may exert strong antioxidant effects.

### 3.7. Thiamine and Riboflavin Content

Mushrooms are an excellent source of B vitamins, including vitamins B1 (thiamine) and B2 (riboflavin) [67]. In the analyzed fresh mushroom samples, the *A. bisporus* had the highest content of vitamin B1 and B2 (0.44 and 4.52 mg/100 g DW, respectively), whereas the lowest levels of these vitamins were determined in the *P. ostreatus* samples (0.1 and 1.39 mg/100 g DW, respectively) (Table 5).

Similar levels of vitamins B1 and B2 (0.6 and 5.1 mg/100 g DW, respectively) in mushroom fruiting bodies were reported by Muszyńska et al. [68]. Mattila et al. [69] and Furlani et al. [70] confirmed that *A. bisporus* fruiting bodies are a better source of thiamine and riboflavin than *P. ostreatus* and *L. edodes* mushrooms. Substantially lower amounts of thiamine and riboflavin were detected in the fermented mushrooms than in the fresh samples, except the *L. edodes* samples, where the vitamin B1 content remained unchanged. Only two studies were found to analyze the content of B vitamins in fermented mushrooms [71,72]. The researchers compared the effects of different blanching methods on the chemical composition of fermented *A. bisporus*, bay bolete (*Imleria badia*), and red pine mushrooms (*Lactarius deliciosus*). The heat treatment type applied exerted a significant effect on the vitamin content in the mushrooms, and the effect of the treatment was significantly dependent on the mushroom species. In *A. bisporus*, the vitamin B1 content was determined at 0.65–0.86 mg/100 g DW; it was higher in mushrooms blanched in water for 30 s and 2 min than in the microwave-assisted blanching variant. The vitamin B2 content in fermented mushrooms was in the range of 0.47–4.27 mg/100 g DW and was higher in mushrooms blanched for 2 min than in those blanched for 30 s. Unfortunately, no unprocessed mushroom fruiting bodies were analyzed in these studies.

Vitamins B are water-soluble and sensitive to high temperatures, which may explain their loss during processing [70]. Fermented mushrooms are subjected to hydrothermal treatment, but this process takes a relatively short time. No studies have been conducted to date to compare the nutritional value of fermented mushrooms with that of commonly available marinated or sterilized mushroom preserves. However, as reported by Jaworska et al. [73], frozen mushrooms blanched at 96–98 °C for 3 min (as in the case of fermented mushrooms) contain on average 3.5-fold higher content of thiamine and approximately 25% more riboflavin than canned mushrooms sterilized at 118–121 °C for 12 min.

### 3.8. Sensory Evaluation

Mushrooms are highly valued food products due to their organoleptic qualities, primarily the flavor and aroma they impart to mushroom-containing dishes [13,28]. A 9-point hedonic scale was used to evaluate the sensory parameters of fermented mushrooms, with 9 representing the highest score for the assessed characteristic. The color, aroma, flavor, and texture of the finished products were assessed. The highest scores for the flavor, color, and texture were given to the fermented *A. bisporus* (7.91, 7.57, and 8.04, respectively). In terms of the first two quality characteristics, the differences between the individual mushroom species were statistically insignificant (Table 6). The highest score for the aroma was achieved by the fermented *L. edodes* samples (7.78). All the scores ranged from 6.83 (texture of fermented *L. edodes*) to 8.04 (texture of fermented *A. bisporus*), which indicates that fermented mushrooms are perceived as an attractive product by consumers.

As reported by the panelists, the texture of the fermented mushrooms resembles that of marinated mushrooms, but the former have a more delicate taste and a fuller aroma with perceptible mushroom notes. Noteworthy, the recipe used in this experiment did not include any addition of aromatic spices, which may ensure higher scores in the organoleptic evaluation. This was demonstrated by Jabłońska-Ryś et al. [13] in their study, where one of the experimental combinations was supplemented with ground black pepper, bay leaves, and onion slices at 0.1%, 0.2%, and 5%, respectively, of mushroom weight. The addition of the spices largely improved the aroma and flavor of fermented *A. bisporus*.

## 4. Conclusions

The study on the nutritional value of mushrooms has shown that the lactic acid fermentation process significantly reduces the amount of digestible carbohydrates (even up to 7–8 times in the case of *A. bisporus* and *L. edodes*) and the caloric value. Fermented mushrooms can be a valuable source of protein, with its content ranging from 25.5 to 35.1% DW, and fiber, determined at 35.8 to 47.9% DW, in the human diet. Both fresh and fermented mushrooms contain a complete set of essential amino acids, with their highest sum in *A. bisporus* (111.15 mg/g DW and 91.72 mg/g DW in fresh and fermented fruiting bodies, respectively). Mushrooms contain low levels of fat, and their fatty acid profile depends on the species. In the case of unprocessed and lactic acid-fermented *A. bisporus*, the ratio of polyunsaturated fatty acids to saturated fatty acids (over 2:1) is very favorable. The lactic acid fermentation of the *P. ostreatus* and *L. edodes* mushrooms improved the fatty acid profile, significantly increasing the percentage of PUFAs. Mushrooms are a good source of essential omega-6 fatty acids, mainly linoleic acid. Mushrooms fermented by the *L. plantarum* EK11 strain are safe in terms of their biogenic amine content. Only spermidine, with its content ranging from 0.6 to 2.56 mg/g DW, was detected in all the analyzed samples. Various recent research reports indicate the use of spermidine-rich foods for the treatment and prevention of age-related diseases. The role of mushrooms in the diet in this regard seems to be an interesting issue to be determined in further research.

The lactic acid fermentation process not only influences the chemical composition of preserved raw materials but also significantly determines the sensory characteristics of products. The present organoleptic evaluation has shown that fermented mushrooms, with organoleptic scores ranging from 6.83 to 8.04 on a 9-point scale, are products with high consumer acceptance. Lactic acid fermentation is an effective method for preservation of mushrooms; it can be used to increase the microbiological stability and nutritional quality of mushrooms and extend their shelf life. Based on the present results, the *L. plantarum* EK11 strain can be recommended as a suitable starter culture for lactic acid fermentation of mushroom fruiting bodies.

## Figures and Tables

**Table 1:** Nutritional (DW%) and energy value (kcal/100 g DW) of raw and fermented mushrooms.

Proximate	AbF	AbLF	PoF	PoLF	LeF	LeLF
Protein	35.8 ^c^ ± 0.8	35.1 ^c^ ± 0.2	29.5 ^b^ ± 0.5	25.5 ^a^ ± 0.5	37.5 ^d^ ± 0.5	26.0 ^a^ ± 0.5
Fat	3.4 ^c^ ± 0.1	4.2 ^d^ ± 0.1	3.0 ^b^ ± 0.0	2.4 ^a^ ± 0.0	2.3 ^a^ ± 0.0	3.1 ^b^ ± 0.0
Digestible carbohydrates	17.2 ^c^ ± 1.2	2.3 ^a^ ± 1.1	8.5 ^b^ ± 1.5	1.9 ^a^ ± 0.6	10.6 ^b^ ± 2.7	1.2 ^a^ ± 0.4
Total fiber	33.3 ^a^ ± 0.3	35.8 ^a^ ± 1.3	49.9 ^c^ ± 2.1	47.9 ^c^ ± 1.0	42.2 ^b^ ± 2.1	46.3 ^c^ ± 0.2
Ash	10.4 ^c^ ± 0.1	22.7 ^e^ ± 0.0	9.1 ^b^ ± 0.1	22.3 ^d^ ± 0.1	7.4 ^a^ ± 0.0	23.5 ^f^ ± 0.1
Energy value	309.0 ^e^ ± 0.3	258.5 ^b^ ± 2.8	278.8 ^c^ ± 3.7	227.2 ^a^ ± 2.6	297.8 ^d^ ± 4.2	229.1 ^a^ ± 0.4

Ab—*Agaricus bisporus*, Po—*Pleurotus ostreatus*, Le—*Lentinula edodes*, F—fresh, LF—lacto-fermented. Values are mean ± SD of replicates (*n* = 3); values in the rows with different letters are significantly different from each other at *p* ≤ 0.05. SD of 0.0 is <0.05.

**Table 2:** Amino acid content (mg/g DW) in raw and fermented mushrooms.

Amino Acids	AbF	AbLF	PoF	PoLF	LeF	LeLF
Aspartic acid	37.57 ^e^ ± 0.59	25.57 ^c^ ± 0.22	15.52 ^a^ ± 0.10	17.41 ^b^ ± 0.20	27.00 ^d^ ± 0.32	16.36 ^a^ ± 0.16
Threonine *	13.32 ^e^ ± 0.38	10.91 ^c^ ± 0.10	5.98 ^a^ ± 0.03	7.29 ^b^ ± 0.02	12.15 ^d^ ± 0.05	7.61 b^b^ ± 0.13
Serine	12.72 ^e^ ± 0.22	9.17 ^c^ ± 0.11	5.65 ^a^ ± 0.05	6.95 ^b^ ± 0.04	11.18 ^d^ ± 0.16	7.20 ^b^ ± 0.08
Glutamic acid	47.78 ^d^ ± 0.81	32.73 ^c^ ± 0.22	18.52 ^a^ ± 0.06	18.96 ^a^ ± 0.06	56.28 ^e^ ± 0.43	29.91 ^b^ ± 0.39
Proline	14.01 ^e^ ± 0.54	10.53 ^d^ ± 0.16	5.16 ^a^ ± 0.03	6.39 ^b^ ± 0.05	8.74 ^c^ ± 0.05	6.41 ^b^ ± 0.18
Glycine	10.82 ^f^ ± 0.19	9.21 ^d^ ± 0.09	5.41 ^a^ ± 0.01	6.38 ^b^ ± 0.02	9.51 ^e^ ± 0.09	6.65 ^c^ ± 0.05
Alanine	19.00 ^e^ ± 0.27	13.86 ^d^ ± 0.16	7.19 ^a^ ± 0.02	8.48 ^b^ ± 0.08	12.27 ^c^ ± 0.16	8.15 ^b^ ± 0.11
Cysteic acid	5.43 ^c^ ± 0.03	5.21 ^b^ ± 0.15	6.61 ^d^ ± 0.03	3.47 ^a^ ± 0.03	7.70 ^e^ ± 0.03	5.19 ^b^ ± 0.06
Valine *	12.51 ^e^ ± 0.32	10.44 ^c^ ± 0.11	5.62 ^a^ ± 0.13	7.07 ^b^ ± 0.03	10.89 ^d^ ± 0.13	7.35 ^b^ ± 0.11
Methionine sulfone *	7.31 ^e^ ± 0.13	5.78 ^c^ ± 0.12	7.89 ^f^ ± 0.15	5.25 ^b^ ± 0.05	7.29 ^d^ ± 0.16	4.51 ^a^ ± 0.06
Isoleucine *	16.79 ^e^ ± 0.32	13.80 ^d^ ± 0.22	6.04 ^a^ ± 0.03	8.74 ^b^ ± 0.24	13.67 ^d^ ± 0.06	10.74 ^c^ ± 0.41
Leucine *	18.37 ^e^ ± 0.38	16.91 ^d^ ± 0.10	8.39 ^a^ ± 0.44	11.05 ^b^ ± 0.06	14.64 ^c^ ± 0.17	11.61 ^b^ ± 0.16
Tyrosine	9.24 ^e^ ± 0.13	6.81 ^d^ ± 0.07	3.57 ^a^ ± 0.06	4.25 ^b^ ± 0.05	5.38 ^c^ ± 0.13	4.01 ^b^ ± 0.05
Phenylalanine *	14.01 ^d^ ± 0.32	11.95 ^c^ ± 0.17	9.10 ^b^ ± 0.06	7.84 ^a^ ± 0.01	11.99 ^c^ ± 0.22	8.23 ^a^ ± 0.09
Histidine *	7.25 ^d^ ± 0.14	6.10 ^c^ ± 0.11	3.31 ^a^ ± 0.02	3.87 ^b^ ± 0.03	5.96 ^c^ ± 0.04	4.05 ^b^ ± 0.05
Lysine *	15.46 ^e^ ± 0.27	13.26 ^d^ ± 0.11	6.79 ^a^ ± 0.01	8.72 ^b^ ± 0.07	13.50 ^d^ ± 0.22	9.28 ^c^ ± 0.15
Arginine	11.12 ^d^ ± 0.22	10.81 ^d^ ± 0.11	6.41 ^a^ ± 0.05	8.40 ^c^ ± 0.06	12.52 ^e^ ± 0.11	7.73 ^b^ ± 0.04
Tryptophan *	6.13 ^f^ ± 0.04	2.57 ^d^ ± 0.08	1.44 ^a^ ± 0.03	1.61 ^b^ ± 0.10	4.05 ^e^ ± 0.06	1.86 ^c^ ± 0.02
Σ AA	278.86 ^f^ ± 4.99	215.62 ^d^ ± 2.16	128.61 ^a^ ± 0.49	142.13 ^b^ ± 0.16	244.73 ^e^ ± 2.16	156.83 ^c^ ± 2.13
Σ EAA	111.15 ^e^ ± 2.03	91.72 ^d^ ± 0.90	54.57 ^a^ ± 0.43	61.43 ^b^ ± 0.37	94.14 ^d^ ± 0.85	65.23 ^c^ ± 1.10

*—essential amino acids, Ab—*Agaricus bisporus*, Po—*Pleurotus ostreatus*, Le—*Lentinula edodes*, F—fresh, LF—lacto-fermented, Σ AA—sum of amino acids, Σ EAA—sum of essential amino acids. Values are mean ± SD of replicates (*n* = 3); values in the rows with different letters are significantly different from each other at *p* ≤ 0.05.

**Table 3:** Biogenic amine content (mg/g DW) in raw and fermented mushrooms.

Biogenic Amines	AbF	AbLF	PoF	PoLF	LeF	LeLF
Cadaverine	ND	ND	ND	ND	ND	ND
Putrescine	ND	ND	ND	ND	ND	ND
Tyramine	ND	ND	ND	ND	ND	ND
Histamine	ND	ND	ND	ND	ND	ND
Spermine	ND	ND	ND	ND	ND	ND
Spermidine	2.56 ^c^ ± 0.42	1.68 ^b^ ± 0.02	0.69 ^a^ ± 0.00	0.60 ^a^ ± 0.01	1.53 ^b^ ± 0.02	0.87 ^a^ ± 0.01
Agmatine	ND	ND	ND	ND	ND	ND

Ab—*Agaricus bisporus*, Po—*Pleurotus ostreatus*, Le—*Lentinula edodes*, F—fresh, LF—lacto-fermented. Values are mean ± SD of replicates (*n* = 3); values in the rows with different letters are significantly different from each other at *p* ≤ 0.05. SD of 0.00 is <0.005. ND—not detected.

**Table 4:** Fatty acid profile (%) in raw and fermented mushrooms.

Fatty Acids	AbF	AbLF	PoF	PoLF	LeF	LeLF
C6:0	0.28 ^b^ ± 0.03	0.38 ^c^ ± 0.05	0.56 ^d^ ±0.04	0.07 ^a^ ± 0.00	0.80 ^e^ ± 0.03	0.34b ^c^ ± 0.02
C8:0	0.21 ^b^ ± 0.03	0.63 ^d^ ± 0.04	0.36 ^c^ ± 0.01	0.03 ^a^ ± 0.01	1.99 ^e^ ± 0.04	0.58 ^d^ ± 0.02
C10:0	0.31 ^b^ ± 0.04	1.71 ^d^ ± 0.06	0.88 ^c^ ± 0.02	0.02 ^a^ ± 0.00	7.40 ^f^ ± 0.10	1.95 ^e^ ± 0.02
C12:0	0.25 ^b^ ± 0.02	0.74 ^d^ ± 0.01	0.49 ^c^ ± 0.04	0.04 ^a^ ± 0.00	3.32 ^f^ ± 0.02	1.01 ^e^ ± 0.00
C14:0	0.90 ^b^ ± 0.00	1.82 ^d^ ± 0.01	1.48 ^c^ ± 0.10	0.34 ^a^ ± 0.01	6.89 ^f^ ± 0.01	2.74 ^e^ ± 0.03
C15:0	0.46 ^a^ ± 0.01	0.52 ^b^ ± 0.00	0.69 ^c^ ± 0.01	1.13 ^e^ ± 0.01	1.03 ^d^ ± 0.00	1.03 ^d^ ± 0.00
C16:0	16.65 ^c^ ± 0.31	15.17 ^b^ ± 0.09	20.31 ^e^ ± 0.19	14.50 ^a^ ± 0.10	23.65 ^f^ ± 0.03	18.48 ^d^ ± 0.09
C16:1n7	0.67 ^c^ ± 0.02	0.39 ^a^ ± 0.02	1.85 ^e^ ± 0.07	0.80 ^d^ ± 0.01	0.51 ^b^ ± 0.03	0.50 ^b^ ± 0.00
C17:0	0.40 ^c^ ± 0.05	0.35 ^c^ ± 0.00	0.18 ^b^ ± 0.00	0.10 ^a^ ± 0.00	0.41 ^c^ ± 0.02	0.22 ^b^ ± 0.02
C18:0	5.33 ^b^ ± 0.15	5.35 ^b^ ± 0.14	6.11 ^d^ ± 0.04	2.03 ^a^ ± 0.01	12.79 ^e^ ± 0.01	5.84 ^c^ ± 0.01
C18:1n9c + C18:1n9t	7.61 ^b^ ± 0.07	3.55 ^a^ ± 0.01	30.79 ^e^ ± 0.05	20.97 ^d^ ± 0.11	11.26 ^c^ ± 0.09	7.45 ^b^ ± 0.03
C18:2n6c + C18:2n6t	63.07 ^d^ ± 0.39	65.10 ^e^ ± 0.22	32.50 ^b^ ± 0.30	54.21 ^c^ ± 0.02	26.33 ^a^ ± 0.20	53.98 ^c^ ± 0.01
C18:3n6 (γ)	ND	ND	ND	ND	0.13 ^a^ ± 0.01	0.29 ^b^ ± 0.00
C18:3n3 (α)	ND	ND	0.04 ^a^ ± 0.01	0.09 ^b^ ± 0.00	0.04 ^a^ ± 0.01	0.08 ^b^ ± 0.00
C20:0	1.66 ^b^ ± 0.06	1.78 ^c^ ± 0.04	ND	0.24 ^a^ ± 0.01	0.32 ^a^ ± 0.02	0.25 ^a^ ± 0.02
C20:1n9	ND	ND	ND	0.14 ^b^ ± 0.00	0.21 ^c^ ± 0.02	0.10 ^a^ ± 0.00
C20:3n6	0.12 ^a^ ± 0.04	0.09 ^a^ ± 0.00	ND	ND	ND	ND
C20:5n3	1.13 ^d^ ± 0.05	1.00 ^c^ ± 0.05	0.27 ^ab^ ± 0.01	0.29 ^b^ ± 0.01	0.21 ^ab^ ± 0.01	0.19 ^a^ ± 0.01
C22:0	ND	ND	ND	0.14 ^b^ ± 0.01	ND	0.07 ^a^ ± 0.01
C22:1n9	0.50 ^bc^ ± 0.02	0.31 ^ab^ ± 0.12	0.38 ^ab^ ± 0.06	0.25 ^ab^ ± 0.08	0.74 ^c^ ± 0.11	0.22 ^a^ ± 0.13
C22:2n6	ND	ND	ND	0.07 ^a^ ± 0.01	ND	0.07 ^a^ ± 0.00
C23:0	0.45 ^a^ ± 0.02	1.12 ^b^ ± 0.02	2.55 ^d^ ± 0.30	3.56 ^e^ ± 0.14	1.97 ^c^ ± 0.28	4.61 ^f^ ± 0.18
C22:6n3	ND	ND	0.56 ^a^ ± 0.14	0.97 ^b^ ± 0.00	ND	ND
SFA	26.90 ^b^ ± 0.45	29.56 ^c^ ± 0.15	33.61 ^d^ ± 0.08	22.20 ^a^ ± 0.03	60.56 ^f^ ± 0.45	37.11 ^e^ ± 0.17
MUFA	8.78 ^c^ ± 0.03	4.25 ^a^ ± 0.13	33.02 ^f^ ± 0.16	22.16 ^e^ ± 0.04	12.72 ^d^ ± 0.25	8.27 ^b^ ± 0.16
PUFA	64.32 ^e^ ± 0.48	66.19 ^f^ ± 0.27	33.37 ^b^ ± 0.15	55.63 ^d^ ± 0.01	26.71 ^a^ ± 0.21	54.62 ^c^ ± 0.01
Omega 3	1.13 ^c^ ± 0.05	1.00 ^bc^ ± 0.05	0.87 ^b^ ±0.15	1.35 ^d^ ± 0.00	0.25 ^a^ ± 0.00	0.27 ^a^ ± 0.01
Omega 6	63.19 ^d^ ± 0.43	65.19 ^e^ ± 0.22	32.50 ^b^ ± 0.30	54.28 ^c^ ± 0.01	26.46 ^a^ ± 0.20	54.34 ^c^ ± 0.00
Omega 9	8.11 ^b^ ± 0.05	3.85 ^a^ ± 0.11	31.17 ^e^ ± 0.10	21.36 ^d^ ± 0.03	12.21 ^c^ ± 0.21	7.77 ^b^ ± 0.16

Ab—*Agaricus bisporus*, Po—*Pleurotus ostreatus*, Le—*Lentinula edodes*, F—fresh, B—blanched, LF—lacto-fermented. SFA—saturated fatty acid, MUFA—mono unsaturated fatty acid, PUFA—poly unsaturated fatty acid. Values are mean ± SD of replicates (*n* = 3); values in the rows with different letters are significantly different from each other at *p* ≤ 0.05. SD of 0.00 is <0.005. ND—not detected.

**Table 5:** Thiamine and riboflavin content (mg/100 g DW) in raw and fermented mushrooms.

Vitamine	AbF	AbLF	PoF	PoLF	LeF	LeLF
Thiamine	0.44 ^d^ ± 0.02	0.08 ^b^ ± 0.00	0.10 ^b^ ± 0.01	0.02 ^a^ ± 0.00	0.30 ^c^ ± 0.02	0.29 ^c^ ± 0.01
Riboflavin	4.52 ^f^ ± 0.02	2.11 ^e^ ± 0.02	1.39 ^c^ ± 0.04	0.28 ^a^ ± 0.01	1.90 ^d^ ± 0.02	0.52 ^b^ ± 0.01

Ab—*Agaricus bisporus*, Po—*Pleurotus ostreatus*, Le—*Lentinula edodes*, F—fresh, LF—lacto-fermented. Values are mean ± SD of replicates (*n* = 3); values in the rows with different letters are significantly different from each other at *p* ≤ 0.05. SD of 0.00 is <0.005.

**Table 6:** Sensory evaluation of fermented mushrooms.

Samples	Color	Aroma	Taste	Texture
AbLF	7.57 ^a^ ± 1.04	7.17 ^ab^ ± 1.07	7.91 ^a^ ± 0.9	8.04 ^b^ ± 0.77
PoLF	7.17 ^a^ ± 1.3	6.87 ^a^ ± 1.14	7.17 ^a^ ± 1.44	6.96 ^a^ ± 1.15
LeLF	7.00 ^a^ ± 1.48	7.78 ^b^ ± 1.28	7.61 ^a^ ± 1.23	6.83 ^a^ ± 1.27

AbLF—lacto-fermented *Agaricus bisporus*, PoLF—lacto-fermented *Pleurotus ostreatus*, LeLF—lacto-fermented *Lentinula edodes*. Values are mean ± SD of replicates (*n* = 23); values in the columns with different letters are significantly different from each other at *p* ≤ 0.05.

## Data Availability

The original contributions presented in the study are included in the article, further inquiries can be directed to the corresponding author.
